# Revisiting interactions between polygalacturonases and polygalacturonase-inhibiting proteins and their effects on plant health: a review

**DOI:** 10.3389/fpls.2025.1691420

**Published:** 2025-11-03

**Authors:** Pallavi Mallick, Dikchha Singh, Prassan Choudhary, Varaprasad Kolla

**Affiliations:** ^1^ Amity Institute of Biotechnology, Amity University, Raipur, Chhattisgarh, India; ^2^ CSIR -Central Institute of Mining and Fuel Research, Dhanbad, Jharkhand, India; ^3^ Academy of Scientific and Innovative Research (AcSIR), Ghaziabad, India

**Keywords:** polygalacturonases, polygalacturonase inhibiting proteins, machine learning, pectinolytic, ecological monitoring

## Abstract

Polygalacturonases (PG) are recognized as key cell-wall-degrading enzymes in phytopathogenic fungi. Plants have a well-developed defense mechanism to counter invasive pathogens, yet such attacks harm the plants. The present review aims to understand the interactions of fungal polygalacturonases with their host invasions affecting plant health. The study also discusses in detail the structure–function relationships of PG interactions with their host counterparts. The role of PGIPs has been emphasized in correlation to algorithms designed to unravel plant microbe interactions involving PGs. With ever-changing environmental stress indicators, it becomes absolutely important to understand plant adaptations on a proteomic and metabolomic level. This would help plant disease diagnosticians in devising early warning or prediction systems for crop-specific protection based on PG–PGIP interactions.

## 1 Introduction

The plant cell wall is a rigid, protective structure that surrounds the plasma membrane, providing mechanical strength and acting as a barrier against external stresses. It is primarily composed of polysaccharides, proteins, and aromatic polymers, with its basic constitution conserved across species. This includes a matrix of pectin embedded with cellulose and hemicellulose microfibrils, along with hemicellulose, lignin, and structural proteins (Kubicek et al., 2014). Despite variations in composition among species, the plant cell wall’s fundamental role in structural integrity and defense remains universal.

As a key barrier against microbial invasion, the plant cell wall is frequently targeted by pathogens. Microbes secrete cell-wall-degrading enzymes (CWDEs) to overcome cell wall defense, causing structural modifications like swelling to facilitate penetration. While CWDEs are not always critical for initial invasion, they play a pivotal role during the later stages of infection, enabling tissue colonization by breaking down cell wall components (Lorrai and Ferrari, 2021). Among these CWDEs, polygalacturonases (PGs), specifically endo-PGs (EC 3.2.1.15) and exo-PGs (EC 3.2.1.67), are of particular importance. These enzymes, produced by fungal pathogens, degrade the pectin network in the plant cell wall, exposing cellulose and hemicellulose for further degradation by hemi-cellulases and cellulases (D’Ovidio et al., 2004; De Lorenzo et al., 2001; Kalunke et al., 2015).

Pectins, which are abundant in the primary cell wall and middle lamellae, play a crucial role in maintaining structural integrity and cohesion in plant tissues. These polysaccharides are rich in negatively charged or methyl-esterified galacturonic acid and are generally found as water-insoluble protopectin. Pectins exist in three main forms: homogalacturonan (a linear chain of galacturonic acid residues), rhamnogalacturonan-I, and rhamnogalacturonan-II (Chandrayan, 2018; Palin and Geitmann, 2012). The main pectin chain comprises “hairy” and “smooth” regions, with the former containing large side chains that require additional accessory enzymes for degradation. Structurally, pectin predominantly consists of two distinct domains: polygalacturonic acid, a homopolymer of α-d-galactosyluronic acid, and rhamnogalacturonan, a heteropolymer with repeating disaccharides of rhamnosyl and galactosyluronic acid (Nakamura and Iwai, 2019; Pedrolli et al., 2009).

PGs which hydrolyze the α-(1–4) linkages in homogalacturonan are produced by plants, microbes such as fungi and bacteria, as well as insects and nematodes (Gadre et al., 2003; Kalunke et al., 2015; Kubicek et al., 2014). During cell wall degradation by PGs, oligogalacturonides (OGs) are released, which act as damage-associated molecular patterns (DAMPs). These OGs trigger the plant’s innate immune responses, including oxidative bursts, phytoalexin accumulation, and the activation of pathogenesis-related genes (De Lorenzo et al., 2001; Nakamura and Iwai, 2019).

To counteract the activity of PGs, plants produce polygalacturonase-inhibiting proteins (PGIPs), which are localized in the cell wall and endomembrane system. PGIPs inhibit fungal PGs through protein–protein interactions, effectively reducing their hydrolytic activity. These proteins specifically recognize PGs released by pathogens and pests such as fungi, oomycetes, insects, and nematodes. PGIP gene expression is induced by pathogens and various phytohormones, including abscisic acid (ABA), indole-3-acetic acid (IAA), salicylic acid (SA), and jasmonic acid (JA) (Hou et al., 2015, 2019; Lorrai and Ferrari, 2021; Lim et al., 2015). Interestingly, PGIPs do not bind to plant-derived PGs but interact with fungal PGs and pectin components of the cell wall (Howell and Davis, 2005; Yang et al., 2021).

PGIPs play a dual role in plant defense: they restrict fungal progression by inactivating PGs and promote the release of OGs, which further activate plant immune responses. Structurally, PGIPs are leucine-rich repeat (LRR) proteins, characterized by 10 modified repeats of 24 amino acid leucine-rich peptides (De Lorenzo et al., 2001; Kalunke et al., 2015). These proteins not only delay cell wall degradation but also enhance the persistence of OGs, prolonging their signaling role in defense activation (Cervone et al., 1986). The discovery of PGIP gene activity dates back to 1971 by Albersheim and Anderson (Albersheim and Anderson, 1971; Jones et al., 1972). The interaction between PGs and PGIPs will be discussed in greater detail in the following sections.

## 2 Polygalacturonases

**Figure 1:**
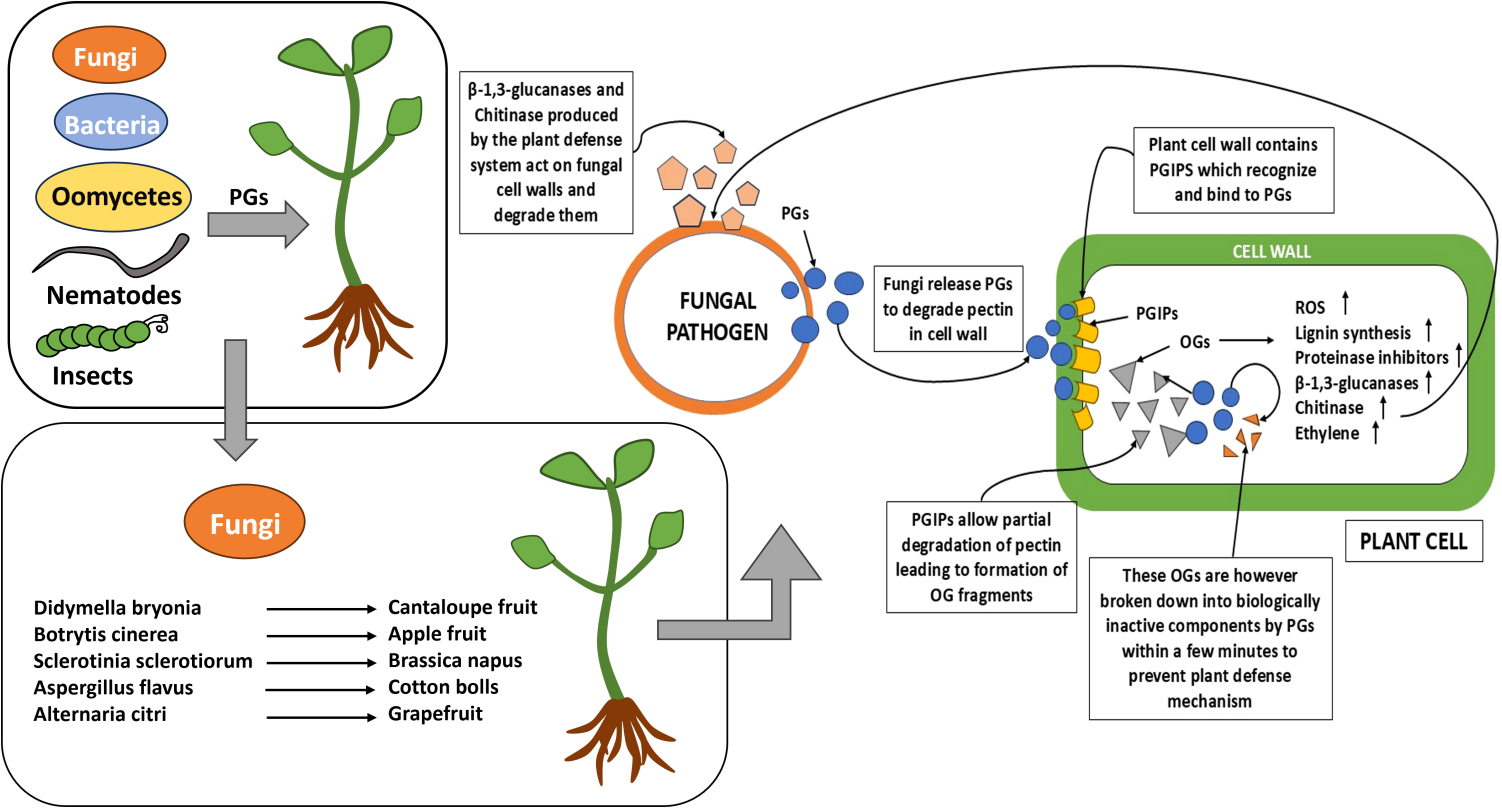
The diagram depicts the various biotic stress factors involved in host–pathogen interactions.

Polygalacturonase is an enzyme produced in microbes and nematode which hydrolyzes plant cell wall, especially pectin network, leading to disease progression. There are many microbial infections known to be produced through this mechanism, mostly fungal infection. PG, also known as pectolytic glycanase, is produced in fungi and helps fungi to penetrate plants, demolishing the plant’s first defense barrier and hence causing infection (**Figure 1**). PGs are also produced by plants itself and helps in fruit ripening, cell separation, and pollen tube growth.

### 2.1 Structure and factors affecting PGs

The structure of polygalacturonase (PG) is found to be a 349-amino-acid enzyme, revealing right-handed β-helix architecture from *Fusarium moniliforme* (FmPG). This structure is composed of 10 layers of parallel β-sheets (PB1, PB2a, PB2b, and PB3), with PB2a being a unique feature of PG (Safran, 2021). The active site cleft, open at both the N- and C-termini, resides in a groove formed by connecting loops and contains six critical residues: H188, R267, and K269 for substrate binding, D212 for proton donation, and D213 and D191 for water activation during nucleophilic attack. Structural stability is maintained through multiple factors, including hydrophobic core interactions, four disulfide bridges, an α-helix at the N-terminus, and a flexible loop at the C-terminus. Key residues, such as Lys-269, Arg-267, and His-188, play essential roles in forming a stable enzyme–substrate complex, enabling effective substrate binding and inhibition (Cea et al., 2024). Catalytic residues, including Asp-191, Asp-212, and Lys-269, are critical for enzymatic function and inhibition. Structural comparisons between FmPG and *Aspergillus niger* polygalacturonase (AnPGII) underscore the significance of conserved residues in polygalacturonase-inhibiting protein (PGIP) recognition sites (Bonivento et al., 2008; Federici et al., 2001). Interestingly, plant PGs lack these key residues, preventing interactions with PGIPs. This structural distinction ensures that PGIPs specifically target fungal PGs while avoiding interference with plant pectin remodeling processes, which are crucial for growth, development, and wound repair (Petrasch, 2020).

Although the cleft structure has minimal impact on PG enzymatic activity, it is essential for PGIP recognition. Unlike universally conserved amino acids directly linked to pathogenicity, each PG involved in pathogenicity or virulence exhibits a unique structure adapted to the complex pectic substances of specific plant species. Additionally, the presence, absence, or type of N-terminal extension in PGs represents a critical structural feature influencing substrate specificity and mediating interactions with distinct regions of the pectin polymer (D’Ovidio et al., 2004; Nakamura and Iwai, 2019; Varland et al., 2015). PGs from an *F. moniliforme* isolate have been found to evade PGIP inhibition due to specific amino acid substitutions. PGs encoded by the pgIII and lpgII genes were not recognized by PGIP, unlike those from pgI. Key differences included a short segment of substitutions in the N-terminal region and five additional substitutions, two of which are near the active site. These changes, located outside the active site cleft, disrupted PGIP recognition. This highlights the critical role of non-active-site regions in PG–PGIP interactions and PGIP resistance (Sella et al., 2004).

The polygalacturonic acid in plant tissues is difficult to degrade just by the action of PGs as it depends on factors like its polymerized structure that contain partially methyl esterified and intricately combined with other polysaccharides. Hence, PGs require the prior action of pectin methylesterases (PMEs; EC 3.1.1.11) to de-esterify pectin, as PGs can only hydrolyze pectate and not the original highly esterified form of pectin. Before PME activity occurs, the degradation of water-insoluble pectin is facilitated by proto-pectinase (PP) activity, which releases soluble pectin from protopectin (Jayani et al., 2005; Nakamura and Iwai, 2019). Another factor influencing the expression of PGs is pH-dependent process which is regulated by proteins such as PacC in *Aspergillus niger* (D’Ovidio et al., 2004). In *Sclerotinia sclerotiorum*, the pH function requires the production of oxalic acid as well as a pH regulator protein Pac1 which is known to be a functional homolog of PacC of *A. niger* (Govrin and Levine, 2000; Rollins and Dickman, 2001).

Oxalic acid enhances the pathogenicity through multiple mechanisms. It acidifies plant tissue, chelates cell wall Ca²^+^ which compromises the function of Ca²^+^-dependent defense responses and also weakens the plant cell wall, and facilitates cell wall degradation by activating enzymes like endo-PG. By acidifying the middle lamella and forming calcium oxalate crystals, oxalic acid weakens cell wall integrity, enabling synergistic action with depolymerizing enzymes (Hegedus and Rimmer, 2005). At pH levels below 3.8, oxalic acid upregulates endo-PG gene expression, boosting enzyme activity and enhancing pectin degradation. It also neutralizes plant defense mechanisms by destabilizing PGIP–PG interactions, rendering PGIPs ineffective (Favaron et al., 2004; Liu et al., 2018; Shi et al., 2009). Liu et al. reported mutant CkPGIP1 and GhPGIP1 genes led to improved resistance against verticillium wilt in cotton (Liu et al., 2018). Oxalic acid suppresses the host’s oxidative burst, reducing ROS and preventing fungal PCD, thereby weakening plant defenses like the hypersensitive response (HR). This paradoxically promotes necrotic tissue formation, which serves as a nutrient source for necrotrophic fungi. Additionally, by lowering the pH, oxalic acid optimizes PG activity, alters signaling pathways such as cAMP regulation, and creates favorable conditions for infection and nutrient acquisition (Cessna et al., 2000; Zuppini et al., 2005). Elevated ROS levels are often correlated with severe infection, highlighting the role of oxalic acid in pathogenic success (Govrin and Levine, 2000).

### 2.2 PGs from pathogen related to plant diseases

Fungal pathogens, including necrotrophs, saprotrophs, biotrophs, and hemibiotrophs, secrete polygalacturonases (PGs) to varying degrees. While PGs are traditionally recognized as virulence factors due to their enzymatic activity, they may also contribute to virulence through non-catalytic mechanisms. Furthermore, many cell-wall-degrading enzymes (CWDEs), including PGs, act as pathogen-associated molecular patterns (PAMPs) that can trigger plant immune responses (**Table 1**). The expression of PGs is induced by pectin, polygalacturonic acid, and metabolic end-products structurally related to their substrates, but it is repressed in the presence of carbon catabolites such as glucose (Annis and Goodwin, 1997; Doehlemann et al., 2017).

**Table 1:** The detailed list of PG genes involved in various plant diseases being caused in agriculturally important crops.

Name of pathogen	PG gene	Type of disease caused	Plants in which the disease is caused	Parts of the plant affected by the disease	References
*Alternaria* *macrospora*	amPG	Boll rot	Cotton	Cotton bolls	Murmu et al., 2025
*Xanthomonas citri* pv. *malvacearum*	xcPG	Bacterial leaf blight	Cotton	Leaf	Murmu et al., 2025
*Geotrichum citri-aurantii*	–	Sour rot	Satsuma mandarin	Fruit	Ai et al., 2025
*Sclerotinia sclerotiorum*	SsPG1, SsPG3, SsPG5, SsPG6, SsXPG1, SsXPG2	Stem rot	Indian mustard	Stem	Rai et al., 2024; Zhu et al., 2024
*Ralstonia solanacearum*	pehA, pehB, pehC	Brown rot	Potatoes	Xylem, vascular system	Elshamy et al., 2024
*Monilinia fructicola*	pg1	Brown rot	Nectarine	Stomata, epidermal cells	Astacio et al., 2024
*Botrytis cinerea*	Bcpg1, Bcpg2	Soft rot	Tomato	Fruit	Silva et al., 2023
*Fusarium oxysporum* f. sp. *ginseng*	Pg1, Pg5, Pgx1, Pgx4	Ginseng root rot	*Panax* *ginseng*	Root	Jun et al., 2022
*Colletotrichum lindemuthianum*	clpg1, clpg2	Anthracnose	Common bean	Leaves, twigs, stems, and fruit	da Silva et al., 2022
*Pectobacterium brasiliense*	pehA	Soft rot	Orchids	Leaf	Sheila et al., 2022
*Podosphaera xanthii*	PG1	Powdery mildew	Cucumber	Leaves, buds, young shoots, fruits, and flowers	Tek et al., 2022
*Erwinia carotovora*	1BHE	Rhizome rot	Banana	Rhizome	Kumar et al., 2020
*Rhizoctonia solani*	RsPG2	Sheath blight	Rice	Sheath and leaves	Chen et al., 2017
*Aspergillus niger*	pgxB, pgaI, pgaII, pgaA, pgaC, pgaD, pgaE	Apple rot	Apple	Fruit	Liu et al., 2017
*Penicillium digitatum*	Pdpg2	Green mold	Citrus cultivars	Fruit	Zhang et al., 2013
*Phytophthora capsici*	Pcipg2	*Phytophthora* blight	Pepper	Leaves	Sun et al., 2009
*Geotrichum candidum*	S31PG1	Soft rot	Lemon	Fruit	Nakamura et al., 2003
*Alternaria citri*	Acpg1	Black rot	Citrus cultivars	Stylar or stem end of the fruit	Isshiki et al., 2001
*Pseudomonas solanacearum*	pglA	Bacterial wilting	Tomato	Leaves	Schell et al., 1988
*Botrytis cinerea*	Bcpg1	Soft rot	Apple	Leaves, fruit	Benito et al., 1998
*Aspergillus flavus*	pecA and pecB	Boll rot	Cotton	Cotton bolls	Shieh et al., 1997
*Erwinia chrysanthemi*	ExoPeh	Soft rot	Potato	Tubers	Barras et al., 1994
*Erwinia carotovora*	Peh	Soft rot	Potato	Tubers	Barras et al., 1994

PGs are classified within the glycoside hydrolase family 28 (GH28) and listed in the CAZy database (Markovič and Janeček, 2001; van den Brink and de Vries, 2011), displaying significant variability in structure, activity, substrate specificity, modes of action, and optimal pH. These properties enable pathogens to adapt to diverse environmental conditions, infect a wide range of plant species, and target specific tissues, highlighting the critical role of PGs in pathogen evolution and host specificity (De Lorenzo and Ferrari, 2002).

Genes encoding PGs belong to highly polymorphic families, with fungi producing multiple isozymes that vary in molecular weight, enzymatic properties, and regulatory mechanisms. Some of the genes encoding PGs include sspg1d, sspg3, sspg5, and sspg6 endoPGs and ssxpg1 and ssxpg2 exo-PGs from *Sclerotinia sclerotiorum* (Favaron et al., 2004); PG2 from *Fusarium moniliforme*; Clpg1 from *Colletotrichum lindemuthianum*; pgaI, pgaII, pgC, and PG2 from *Aspergillus niger*; and pecA and pecB from *Aspergillus flavus* (Annis and Goodwin, 1997). This diversity also reflects the complex structure of pectin in plant cell walls and allows fungal pathogens to effectively cleave homogalacturonan in various structural contexts. The wide array of PG isoforms enhances the adaptability of fungal pathogens across different hosts and environmental conditions while also reducing the risk of losing pathogenicity. The functional diversity of PGs arises from the presence of multiple genes and post-translational modifications, such as glycosylation. Glycosylation has been shown to improve enzyme stability, enhance resistance to protease degradation, and optimize enzymatic activity (D’Ovidio et al., 2004).

### 2.3 Mechanism of host infection in plant and OG production

PGs hydrolyze polygalacturonic acid linkages of pectate by adding water across the oxygen bridge in the smooth pectin region (Radha et al., 2019). Some PGs, like BcPG1 from *Botrytis cinerea* and an endo-PG from *Sclerotinia sclerotiorum*, play dual roles in plant–pathogen interactions. They degrade cell walls to enhance pathogenicity while also acting as elicitors by triggering plant defenses, such as increased production of cytosolic Ca²^+^ which induces programmed cell death that requires cytochrome c release and caspase-like protease activation (Reignault et al., 2008; Zuppini et al., 2005).

The distinction between endo and exo mode of PGs lies in their hydrolytic mechanisms. Endo-PGs cleave homogalacturonan randomly along its length, producing a mixture of oligogalacturonides. In contrast, exo-PGs hydrolyze sequentially at the non-reducing end of the polymer, releasing monomers of galacturonic acid. This random cleavage by endo-PGs results in a faster reduction compared to exo-PGs, which gradually degrade the polymer. Some fungi like *F. oxysporum* require PGs that work in an endo/exo manner, in which the cleavage activity is more rapid than just the exo method but slower than the endo method, but they produce OGs that do not elicit plant defense systems (Cook et al., 1999; Di Pietro et al., 2003; Radha et al., 2019). Endo/exo-PGs are capable of a limited destruction mode of pathogenesis such as hemibiotrophy. On the other hand, the endo-PGs tend toward necrotrophism or saprophytism. Thus, such observations are in agreement with the generalization that endo-PGs may be most suitable for hydrolysis (Gadre et al., 2003).

OGs are formed via partial hydrolysis of polygalacturonic acid which act as damage-associated molecular patterns (DAMPs) that act as defense elicitors. While OGs can serve as a carbon source for pathogens, they also function as signaling molecules activating plant defenses. PGIPs limit endo-PG activity, increasing the stability and concentration of biologically active OGs, which prolong plant defense responses. Upon OG detection, plants activate multi-layered defenses like cell wall strengthening via lignin and protein cross-linking, stomatal closure, and production of reactive oxygen species (ROS) like H_2_O_2_ and O_2_^-^, which are antimicrobial and signal systemic acquired resistance (SAR), as well as synthesis of phytoalexins, lignin, pathogenesis-related proteins (e.g., β-1,3 glucanase and chitinase), and proteinase inhibitors (PIs), which degrade fungal cell walls and deter insect feeding. OGs also contribute to the oxidative burst, generating ROS to damage pathogens, mediate hypersensitive response (HR)-related cell death, and signal further defenses. Additionally, OGs facilitate systemic signaling, priming distant tissues for future attacks through SAR, and are formed during both pathogen attacks and mechanical damage (Boller and He, 2009; De Lorenzo and Ferrari, 2002; Federici et al., 2006).

### 2.4 Understanding PG structure and function relationship

The genetic sequence analysis shows that endo-PGI gene has an open reading frame of 1,458 bp and encodes for protein containing 485 amino acids in *Rhodotorula mucilaginosa* (Abd El-Aziz et al., 2023). The secondary structure analysis revealed that endo-PGI gene contains 22 α-helices and four β sheets and 21 α-helices and three β sheets in two different isolates of *Rhodotorula mucilaginosa* with three active binding pockets (Glu198-Arg334-Ala193-Arg331-Trp231-Gly201, Gln360-Asn357-Phe420, and Arg74-Thr26-His73-Ser30-Ser32). The computational study of polygalacturonase (PGII) from *Aspergillus niger* shows folds of right-handed parallel β-helical structure comprising 10 complete turns with dimension 5 Å × 35 Å × 35 Å (Noorbatcha et al., 2011). The average amino acids are 29 per turn, with 4.8-Å rise per turn and four parallel beta sheets PB1, PB2a, PB2b, and PB3. The active residue is conserved sequences, while the catalytic residue is aspartate group (Asp180, Asp201, and Asp202). The comparative study of PGs of *Colletotrichum lupine* revealed that 87.5% amino acid of the active site lies in the most favorable region, whereas 11.1% and 1.4% were in additionally allowed and generously allowed regions of the Ramachandran plot, respectively (Lotter, 2010). All of the four binding pockets were in favorable regions showing acceptable docking scores. The endo-polygalacturonase BiPG28A from *Bispora* sp. was modeled from endo-PG from *Fusarium moniliforme* (1HG8), and after refining and energy optimization, BiPG28A-GalpA4 conformation was generated (Tu et al., 2018). The conserved residues Asn191, Asp193, Asp214, Asp215, His236, Gly237, Arg269, Lys271, and Tyr304 are involved in electrostatic interaction with GalpA, forming four hydrogen bonding resulting in substrate binding (Tu et al., 2018).

The sequence analysis from carbohydrate-active enzymes (CAZy) database and Swiss-Prot database using the CLUSTALW program shows five major clusters: Asn-Thr-Asp, Asp-Asp, Gly-His-Gly, Ser-Ile-Gly-Ser, and Arg-Ile-Lys—indicating functional conservation. Gly residue is conserved in bacterial and plant PG, whereas Gln is found in fungal PG, depicting that the residues differ according to speciation. The polygalacturonase lateral root (PGLR) and *Arabidopsis* dehiscence zone polygalacturonase2 (ADPG2) also has a right-handed parallel β-helical structure (Safran et al., 2023). These have slightly different conformations having three repeating parallel β-sheets—PB1, PB2, and PB3 and small β-sheet, PB1a revealed by computational tools of homology modeling and molecular docking. It contains α-helix at the N-terminus which forms disulphide bridge shielding the hydrophobic core of the enzyme. The modeled structure of the PG gene from two *Arabidopsis* sp. shows four α-helices and 23 β-strands in AtPG12 and five α-helices and 23 β-strands (Both, 2009).

## 3 Plant defensive response relative to polygalacturonases

Plants possess a “cell wall feedback signaling” system that monitors the integrity of the cell wall and activates defense or repair mechanisms upon detecting damage (Liu et al., 2018; Nühse, 2012). Their innate immune system comprises two key branches: pattern-triggered immunity (PTI) and effector-triggered immunity (ETI). PTI is activated when pattern recognition receptors (PRRs) on the cell surface detect conserved microbial sequence patterns of PGs. This recognition initiates broad-spectrum defenses, including receptor-like proteins (RLPs) and receptor-like kinases (RLKs) (Chisholm et al., 2006; Federici et al., 2006; Jones and Dangl, 2006).

Thereby, ETI involves intracellular nucleotide-binding leucine-rich repeat (NLR) proteins that recognize specific pathogen effectors triggering a hypersensitive response (HR), leading to cell death and necrosis (Derbyshire and Raffaele, 2023; Jones and Dangl, 2006; Yang, 2006). The ETI and PTI pathways overlap, providing enhanced protection, particularly against necrotrophic pathogens (Derbyshire and Raffaele, 2023). PGIP (polygalacturonase-inhibiting protein) is one such example of extracellular leucine-rich repeat (LRR) proteins. PGIPs inhibit fungal polygalacturonases, preventing cell wall degradation, and contribute to immune responses being a potent candidate for plant disease resistance (Federici et al., 2001). PGIP’s LRR-RLPs (leucine rich repeat-receptor-like proteins) are known to enhance resistance without triggering hypersensitive responses (McCombe et al., 2022). Although plant defense signaling relies on three key molecules, viz., salicylic acid (SA), jasmonic acid (JA), and ethylene, regulating immune responses, PGIP expression often regulates these phytohormones (Chisholm et al., 2006)—for instance, abscisic acid induces PGIP expression in rice, alfalfa, and pepper, while jasmonic acid and salicylic acid trigger PGIP production in rapeseed, rice, barrel clover, and pepper (Ellur et al., 2023a; Ellur et al., 2023b).

## 4 PGIPs

### 4.1 Structural aspects of PGIPs

PGIPs consist of 10 incomplete LRRs (~24 residues each) arranged into two β-sheets, with β1 on the inner concave side while the opposite side contains nine 3_10_-helices and β2 on the outer convex side. The number of tandem repeats of PGIP varies in different plants, from rice, alfalfa, to wheat plants which possess nine repeats, whereas 11 LRR tandem repeats are present in sugar beet (Rathinam et al., 2020). The LRR motif is structurally crucial for numerous protein–protein interactions due to its adaptability and recognition of various specific targets. Protein conformation in this motif are involved in a wide range of cellular processes, including receptor dimerization, adhesion regulation, domain repulsion, and binding activities (Leckie et al., 1999).

The β-sheet B2 provides an interaction surface, facilitated by unique glycine residues that allow bending. PGIPs, members of the LRR superfamily, function as immune receptors critical for plant immunity and are present in all characterized plant species, serving as defense molecules against fungal pathogens (Upadhyay, 2024). The β-sheet/β-turn region is hypervariable, likely contributing to ligand specificity within this protein class. Structurally, PGIPs consist of an N-terminal domain (residues 1–52) with an α-helix and β-strand, a central LRR domain folded into a right-handed superhelix featuring conserved hydrophobic residues (Leu, Ile, Val, Phe, Tyr), an asparagine ladder forming hydrogen bonds, and a C-terminal domain with 3_10_-helices stabilized by water-mediated hydrogen bonds, a strand of B2, and a loop. The protein’s hydrophobic core is supported by four disulfide bridges, with aromatic residues strategically positioned near the protein’s bend (Di Matteo et al., 2003; Gomathi et al., 2006; Kalunke et al., 2015).

These LRR motifs, containing β-sheet/β-turn/α-helix structures, are essential for interacting with PGs. The bonds that are present help in the overall flexibility for PGIPs to adapt their structure to bind with other proteins. Most PGIPs are generally found to be intronless, except a few that include a short intron. While most PGIPs share a common structure, one particular PGIP that is the CaPGIP2 of chickpea was found to not share this common characteristic which may be the reason for its non-functionality (Ellur et al., 2023b).

### 4.2 Plant PGIP genes and their role in plant defenses

Polygalacturonase-inhibiting proteins (PGIPs) are innate defense proteins localized in the plant cell wall and secreted into the apoplast. It is a glycoprotein with molecular masses ranging from 15 kDa (peach) to 91 kDa (pear), with most between 34 and 54 kDa. PGIPs exhibit competitive inhibition as well as non-competitive inhibition, with PG–PGIP complexes dissociating at pH less than 4.5 and above 6 or in salt concentrations higher than 500 mM sodium-acetate. Their inhibition kinetics and target specificity vary across plant species (Gomathi et al., 2006). The primary role of PGIP is to inhibit the degradation of pectin in the middle lamella by inhibiting PGs (produced by pathogenic bacteria and fungi) (Pérez-Donoso et al., 2010). Basically, it targets polygalacturonase enzymatic activity, playing crucial role in the plant’s structural and immune defense mechanism against microbial invasion. PGIP is a leucine-rich repeat (LRR) protein structure that regulates disease resistance mechanism in plant species by PGIP–gene transfer mechanism (Volpi et al., 2013).

PGIPs bind with the unique surface protein active sites of PGs, anchoring these enzymes to the plant cell wall. This binding leads to the conformational changes of PG which disrupt its activity, thereby preventing the degradation of plant tissue and limiting pathogen spread. They bind with demethylated pectins, which are modified by plant pectin methylesterases (PMEs) and create ideal binding sites (Kumar et al., 2024). During pathogen attacks, PGIPs anchored to these demethylated pectins inhibit fungal PGs by forming enzyme–inhibitor complexes. This not only protects the structural integrity of the cell wall but also triggers the plant’s immune system by facilitating the accumulation of defense-eliciting OGs (De Lorenzo and Ferrari, 2002). Interestingly, PGIPs are ineffective against most pectic enzymes, including those produced by plants. Plants also produce their own pectic enzymes including PGs which are involved in normal developmental processes like cell wall modification, fruit ripening, and response to stress.

The expression of PGIP genes is invariably present in plants, animals, and microorganisms across multiple chromosomes. Due to its importance in plant biochemical anti-disease factor, PGIP is well studied in plants—for instance, in chickpea (*Cicer arietinum*), the genes CaPGIP3 and CaPGIP4 are located on chromosome 3, while CaPGIP1 and CaPGIP2 are present on chromosome 6 (Ellur, 2022; Ellur et al., 2023b). However, only three of these PGIPs (CaPGIP1, CaPGIP3, and CaPGIP4) are functionally active. A total of 17 BnPGIPs are likewise found in multiple chromosomal locus of *Brassica napus* L., a minimum of five PGIPs are discovered on *Phaseolus vulgaris* chr. 10, and AtPGIP1 and AtPGIP2 are two PGIPs present on the same chromosome (chr. 5) of *Arabidopsis thaliana* (Cheng et al., 2021). A total of 41 genes were reported from mungbean (*Vigna radiata*), where four genes (VrPGIP-17, VrPGIP-18, VrPGIP-21, and VrPGIP-23) are found upregulated, resisting bruchid beetle infection (Kumar et al., 2024). BnPGIP2 expression is stimulated by fungal infection, while jasmonic acid regulates it in *Brassica napus*. The elevated expression of AcPGIP was reported in kiwifruit (*Actinidia deliciosa* “Hayward”) with *B. cinerea* infection through proteomic analysis (Guo et al., 2025; H. Liu et al., 2018). The gene ZmPGIP1 from *Zea mays* enhances the mechanical strength of plants, stimulating the production of lignin, cellulose, and hemicellulose in transgenic lines (Xu et al., 2024).

Structurally and functionally, PGIPs share a close relationship with resistance gene (R-gene) products. These R-gene products are believed to act as receptors for pathogen-encoded avirulence (Avr) proteins, playing an integral role for PGIPs in plant defense (Gomathi et al., 2006). Therefore, the regulation of PGIP gene in plants is affected by biotic and abiotic stress conditions enhancing plant defense mechanism. The PGIP downstream gene STOP1 (sensitive to proton rhizotoxicity 1) of *A. thaliana* is dependent on phosphoinositide signaling pathway and independent on nitric oxide signaling, providing aluminum tolerance (Agrahari et al., 2021). Even CsPGIP2 of cucumber, *Cucumis sativus* L., restricts the gray mold of *Botrytis cinerea* from interacting with BcPG3 protein (Jin et al., 2024). The *in silico* study reported that the diverse binding affinity of PGs of *Xanthomonas citri* pv. *malvacearum* and *Alternaria macrospora* with PGIP of cotton (*Gossypium barbadense*) provided a promising approach to develop bacterial leaf blight and leaf spot disease varieties (Murmu et al., 2025). GmPGIP11, a gene from *Glycine max*, is overexpressed during defense, resulting in a decrease in *Heterodera glycines* parasitism in roots (Naskar et al., 2023; Poudel et al., 2024).

PGIPs are remarkably specific and highly adaptable in recognizing a diverse PG pattern, selectively inhibiting fungal and microbial PGs. This specificity is attributed to structural differences between fungal and plant PGs. By targeting mixed-mode (endo/exo) PGs, PGIPs effectively prevent the excessive degradation of plant pectin and prolong the presence of bioactive OGs which are key elicitors in the induction of defense-compound-stimulating plant immune response (Carton et al., 2025). Without the action of PGIPs, these defense-eliciting OGs would be rapidly hydrolyzed into smaller, inactive fragments and monomers, compromising the plant’s ability to mount a strong immune response (Cook et al., 1999; Di Matteo et al., 2006).

### 4.3 Molecular interaction mechanisms of PGIPs

**Figure 2:**
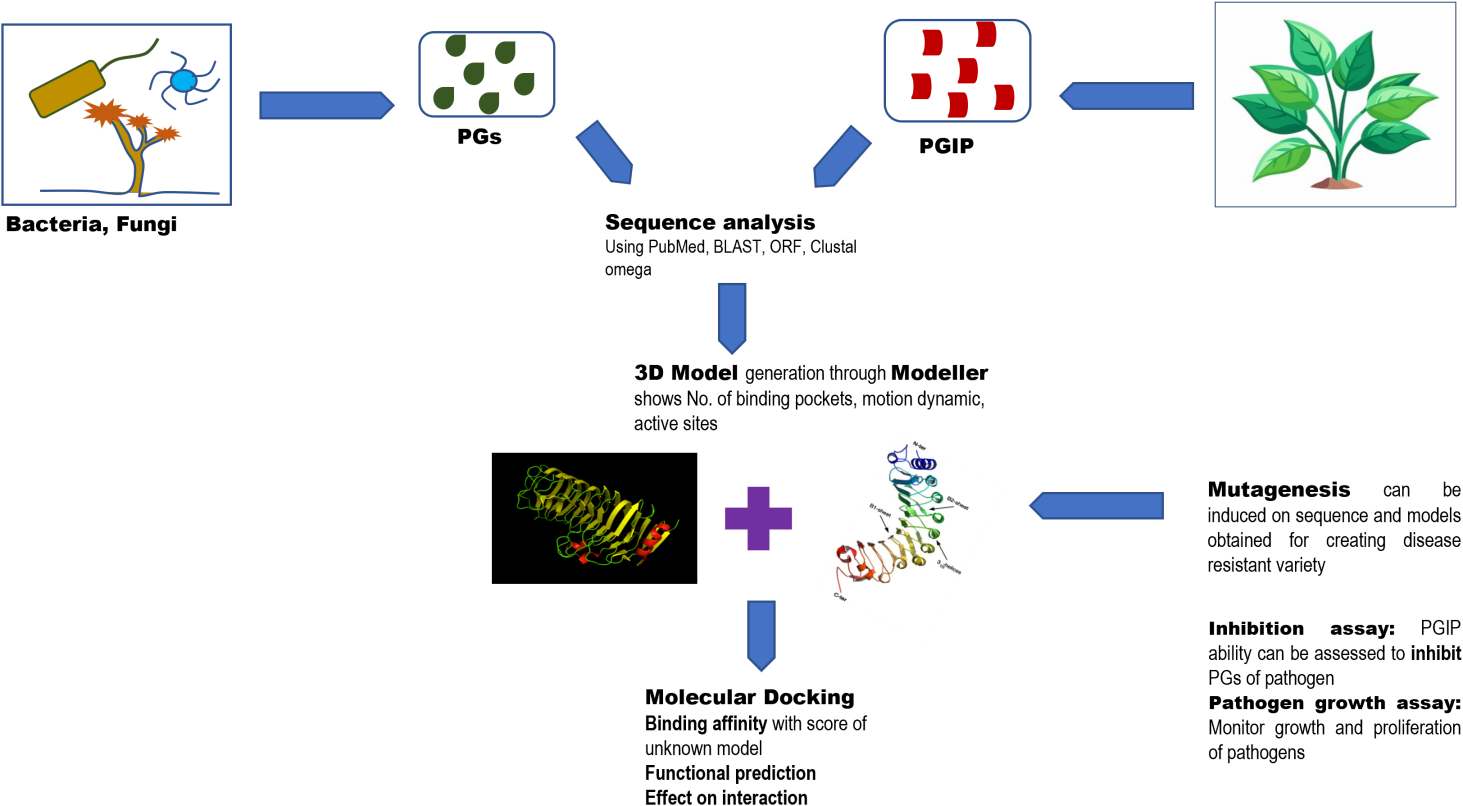
The figure explains the significance of structural understanding of PGIPs to improve the understanding of disease resistance mechanisms involved.

Although PGIPs exhibit a different specificity in their interaction with different polygalacturonase enzymes, the LRR domain is the main interacting site with fungal PGs through both polar and apolar interaction. The PG ligand of this domain possesses a chemically diverse concave face. The binding affinity depends on the orientation of the PG ligand and its inhibition kinetics (**Figure 2**). Depicting this theory, PvPGIP2 of *Phaseolus vulgaris* competitively inhibits the PG of *Fusarium moniliforme* by burying its active site cleft while it non-competitively inhibits *Aspergillus niger* PG by leaving the active site accessible (Federici et al., 2006). Even the PG receptor AtRLP42 in *Arabidopsis thaliana* interacts with specific PG3 motifs (pg9(At) and pg13(At)) stimulating PGIPs to activate pathogen-triggered immunity (PTI) (Derbyshire and Raffaele, 2023). This signal transduction leads to the inhibition of the fungal PG activity to protect cell walls and further triggers the production of pathogenesis-related (PR) proteins, defense metabolites, cell wall remodeling, and programmed cell death (PCD). Diverse PG recognition across species like *Arabidopsis arenosa* and *Brassica rapa* leads to immune robustness in plants.

An interesting study reports the utilization of the method of molecular modeling, docking, and MD simulation for bPGIP–EcPG interaction between banana (*Musa* sp.) and bacteria *Erwinia carotovora* causing soft rot disease (Balamurugan et al., 2020). The interaction of bPGIP and 1BHE are interrelated through whole protein motion dynamics leading to conformational change and imperative signaling. Recently, Chauhan et al. explored PGIP-mediated resistance mechanisms in *M. oleifera* with site-directed mutations E146G and E218K located within the conserved leucine-rich repeat (LRR) domains of moPGIP. The study highlighted the functional relevance of conserved residues in maintaining stable PG–PGIP interactions with enhanced resistance against fungal attacks (Chauhan et al., 2025). A computational study likewise highlighted that GLN242 amino acid mutation by LYS significantly alters the structure and was important for interacting with the PG protein of *Glycine max* against *Sclerotinia sclerotiorum* (Rashmi et al., 2024). Another important study reported that interaction between *Phaseolus vulgaris* PGIP2 (PvPGIP2) and *Fusarium phyllophilum* polygalacturonase (FpPG) enhances substrate binding, resulting in the inhibition of the enzyme activity of FpPG (Xiao et al., 2024). Interestingly, PvPGIP2 binding created a substrate binding site on PvPGIP2–FpPG, forming a new polygalacturonase with boosted substrate binding activity and altered substrate preference. Such studies have highlighted that PGIPs need to alter select amino acid residues to keep pace with everchanging fungal PGs in order to block them. Recent studies on PGIP structures have opened new possibilities of disease resistance studies in plants, thereby aiding plant breeders. Moreover, structural biologists must collaborate with such breeders to understand the dynamic host–pathogen interactions (McClelland and Ma, 2024).

## 5 Role of learning algorithms in understanding PGIP–PGs complex interactions

The structure–function and interaction of PG and PGIP can be studied using many bioinformatics as well as artificial learning approaches. Sequence analysis provides the amino acid sequence of conserved domains and potential binding sites of these proteins. In addition, there are many modeling software that help to form a 3D structure of the sequence extracted of PG and PGIP through which an interaction with binding affinity was experimented and known via molecular docking by understanding the specificity *in silico*. Peng et al. processed heterogeneous (post-) genomics data to understand the role of novel pectinolytic enzymes in *A. niger* (Peng and de Vries, 2021). These approaches are also applied in computational mutagenesis (that is to find the effect of specific amino acid mutation on PG–PGIP interaction) and functional prediction (localization and functional properties of PGIPs). Utilizing the models and algorithm, disease resistance variant is developed, and the evolution and signaling pathway of PGIP and its defense mechanism were better understood. Murmu et al. (2025) noted that different pathogens interact differently to different host PGIPs and mutation on these genes, leading to affect these interactions. The three tools Modeller, Swiss-model, and AlphaFold were utilized for the prediction of *G. barbadense* PGIP (gbPGIP), *A. macrospora* PG (amPG), and *X. citri* pv*. malvacearum* PG (xcPG) 3D model structures, integrating evolutionary, physical, and geometric properties of protein structures to predict their 3D structures (Ragupathy et al., 2023). The generated model with the most negative Z-score is selected and docked for the interaction study using HADDOCK. The estimation of active sites and binding energies provides insights into the stability and specificity of interaction. The molecular dynamics and impact of mutation on cotton (*G. barbadense*) reported in the study provided tools for generation of resistant variants.

Artificial intelligence has aided both structural biology as well as the protein engineering aspects of host–microbe interactions. Studies have proven that PGIP not only acts as a PG receptor but also as an enzymatic manipulator, converting virulence to defense activation (Xiao et al., 2024; Clark et al., 2020). Hence, plant receptors play a dual role in activating immunity. The emergence of artificial intelligence further facilitates protein engineering by offering guidance through *in silico* protein structure prediction (McClelland and Ma, 2024). The crystal structure of the NLR receptor Pikp integrated heavy-metal-associated (HMA) domain in complex with the rice blast fungal effector AVR-Pik which guided the generation of a Pikp mutant that could recognize previously unrecognized AVR-Pik effectors (De la Concepcion et al., 2019). AlphaFold multimer was likewise used to identify interacting residues between the tomato cysteine protease Pip1 and EpiC2B, a protease-inhibiting effector from the potato late blight pathogen *Phytophthora infestans*. Mutagenesis of two targeted amino acids in Pip1 rendered it insensitive to EpiC2B inhibition, and this engineered Pip1 had enhanced resistance to *P. infestans* (Schuster et al., 2024). Algorithms have been designed to understand the RNA–disease interactions in plants (Shi et al., 2024), although much efforts are to be made to understand a plant’s response to various microbial stress especially in cases of fungal pathogens as they might be useful as well as harmful strains may be involved.

## 6 Conclusions

The present review discussed PG–PGIP interactions on molecular, structural, as well as artificial-intelligence-guided studies. Recent studies have shown that protein engineering in PGIP protein structures may provide dual roles to such proteins, thus enhancing the disease resistance capabilities of the host plants. Although recent findings have showed promise, yet fungal PGs have adapted well to the changing environmental stress and remain a great challenge as pathogens. Hence, the future ahead needs collective efforts from structural biologists and plant breeders alike to keep pace with the dynamic host-pathogen interactions keeping in mind the constant threat of environmental stress and epigenomic changes.

## References

[B1] Abd El-Aziz N. M. Moharam M. E. El-Gamal N. N. Khalil B. E. (2023). Enhancement of novel endo-polygalacturonase expression in Rhodotorula mucilaginosa PY18: insights from mutagenesis and molecular docking. Microbial Cell Factories 22, 252. doi: 10.1186/s12934-023-02253-5, PMID: 38066588 PMC10709964

[B2] Agrahari R. K. Enomoto T. Ito H. Nakano Y. Yanase E. Watanabe T.. (2021). Expression GWAS of PGIP1 identifies STOP1-dependent and STOP1-independent regulation of PGIP1 in aluminum stress signaling in Arabidopsis. Front. Plant Sci. 12, 774687. doi: 10.3389/fpls.2021.774687, PMID: 34975956 PMC8719490

[B3] Ai H. Zhang Y. Reymick O. O. Shen X. Liu W. Tao N.. (2025). Extracellular polygalacturonase, β-1,4-glucanase and β-xylosidase in Geotrichum citri-aurrantii positively progressed the sour rot incidence in satsuma mandarin fruit. Postharvest Biol. Technol. 219, 113233.

[B4] Albersheim P. Anderson A. J. (1971). Proteins from plant cell walls inhibit polygalacturonases secreted by plant pathogens. Proc. Natl. Acad. Sci. United States America 68, 1815–1819. doi: 10.1073/pnas.68.8.1815, PMID: 5288769 PMC389299

[B5] Annis S. L. Goodwin P. H. (1997). Recent advances in the molecular genetics of plant cell wall-degrading enzymes produced by plant pathogenic fungi. Eur. J. Plant Pathol. 103, 1–14. doi: 10.1023/A:1008656013255

[B6] Astacio J. D. Melgarejo P. De Cal A. Espeso E. A. (2024). Monilinia fructicola genes involved in the cell wall-degrading process in early nectarine infection. Int. J. Food Microbiol. 419, 110750., PMID: 38776709 10.1016/j.ijfoodmicro.2024.110750

[B7] Balamurugan A. Kumar A. Sakthivel K. Ashajyothi M. Sahu K. P. Karthikeyan M. (2020). Characterization of Dickeya fangzhongdai causing bacterial soft rot disease on Dendrobium nobile in India. Eur. J. Plant Pathol. 158, 773–780. doi: 10.1007/s10658-020-02094-7

[B8] Barras F. van Gijsegem F. Chatterjee A. K. (1994). Extracellular enzymes and pathogenesis of soft-rot Erwinia. Annu. Rev. Phytopathol. 32, 201–234.

[B9] Benito E. P. Ten Have A. van’t Klooster J. W. van Kan J. A. (1998). Fungal and plant gene expression during synchronized infection of tomato leaves by Botrytis cinerea. Eur. J. Plant Pathol. 104, 207–220.

[B10] Boller T. He S. Y. (2009). Innate immunity in plants: an arms race between pattern recognition receptors in plants and effectors in microbial pathogens. Science 324, 742–744. doi: 10.1126/science.1171647, PMID: 19423812 PMC2729760

[B11] Bonivento D. Pontiggia D. Di Matteo A. Fernandez-Recio J. Salvi G. Tsernoglou D.. (2008). Crystal structure of the endopolygalacturonase from the phytopathogenic fungus Colletotrichum lupini and its interaction with polygalacturonase-inhibiting proteins. Proteins: Structure Function Bioinf. 70, 294–299. doi: 10.1002/prot.21610, PMID: 17876815

[B12] Both P. (2009). Structure–function study of Arabidopsis thaliana core alpha1, 3-fucosyltransferase (FucTA) (Université Joseph-Fourier-Grenoble I).

[B13] Carton C. Magnin-Robert M. Randoux B. Pau-Roblot C. Lounès-Hadj Sahraoui A. (2025). Potential of bio-sourced oligogalacturonides in crop protection. Molecules 30, 1392. doi: 10.3390/molecules30061392, PMID: 40142167 PMC11946057

[B14] Cea P. A. Pérez M. Herrera S. M. Muñoz S. M. Fuentes-Ugarte N. Coche-Miranda J.. (2024). Deciphering structural traits for thermal and kinetic stability across protein family evolution through ancestral sequence reconstruction. Mol. Biol. Evol. 41, msae127. doi: 10.1093/molbev/msae127, PMID: 38913681 PMC11229819

[B15] Cervone F. De Lorenzo G. Degra L. Salvi G. (1986). “Interaction of fungal polygalacturonase with plant proteins in relation to specificity and regulation of plant defense response,” in Recognition in microbe-plant symbiotic and pathogenic interactions (Berlin Heidelberg: Springer), 253–258.

[B16] Cessna S. G. Sears V. E. Dickman M. B. Low P. S. (2000). Oxalic acid, a pathogenicity factor for Sclerotinia sclerotiorum, suppresses the oxidative burst of the host plant. Plant Cell 12, 2191–2199. doi: 10.1105/tpc.12.11.2191, PMID: 11090218 PMC150167

[B17] Chandrayan P. (2018). Biological function (s) and application (s) of pectin and pectin degrading enzymes. Biosci. Biotechnol. Res. Asia 15, 87–100. doi: 10.13005/bbra/

[B18] Chauhan D. Behera S. K. Rashmi M. Singh M. K. Shankar R. Jha G. K.. (2025). Functional transformation of Leucine-Rich Repeat (LRR) PolyGalacturonase Inhibitor Protein (PGIP) of Moringa oleifera by dual mutation E146G and E218K in defense against *Cercospora baticola* . Microbe 8, 100519. doi: 10.1016/j.microb.2025.100519

[B19] Chen X. Lili L. Zhang Y. Zhang J. Ouyang S. Zhang Q.. (2017). Functional analysis of polygalacturonase gene RsPG2 from Rhizoctonia solani, the pathogen of rice sheath blight. Eur. J. Plant Pathol. 149, 491–502.

[B20] Cheng S. Li R. Lin L. Shi H. Liu X. Yu C. (2021). Recent advances in understanding the function of the PGIP gene and the research of its proteins for the disease resistance of plants. Appl. Sci. 11, 11123. doi: 10.3390/app112311123

[B21] Chisholm S. T. Coaker G. Day B. Staskawicz B. J. (2006). Host-microbe interactions: shaping the evolution of the plant immune response. Cell 124, 803–814. doi: 10.1016/j.cell.2006.02.008, PMID: 16497589

[B22] Clark K. J. Pang Z. Trinh J. Wang N. Ma W. (2020). Sec-delivered effector 1 (SDE1) of ‘Candidatus Liberibacter asiaticus’ promotes citrus huanglongbing. Mol. Plant-Microbe Interact. 33, 1394–1404. doi: 10.1094/MPMI-05-20-0123-R, PMID: 32986514

[B23] Cook B. J. Clay R. P. Bergmann C. W. Albersheim P. Darvill A. G. (1999). Fungal polygalacturonases exhibit different substrate degradation patterns and differ in their susceptibilities to polygalacturonase-inhibiting proteins. Mol. Plant-Microbe Interact. 12, 703–711. doi: 10.1094/MPMI.1999.12.8.703, PMID: 10432636

[B24] da Silva L. L. Morgan T. Garcia E. A. Rosa R. O. Mendes T. A. de Queiroz M. V. (2022). Pectinolytic arsenal of Colletotrichum lindemuthianum and other fungi with different lifestyles. J. Appl. Microbiol. 133, 1857–1871., PMID: 35766136 10.1111/jam.15692

[B25] De la Concepcion J. C. Franceschetti M. MacLean D. Terauchi R. Kamoun S. Banfield M. J. (2019). Protein engineering expands the effector recognition profile of a rice NLR immune receptor. eLife 8, e47713. doi: 10.7554/eLife.47713, PMID: 31535976 PMC6768660

[B26] D’Ovidio R. Raiola A. Capodicasa C. Devoto A. Pontiggia D. Roberti S.. (2004). Characterization of the complex locus of bean encoding polygalacturonase-inhibiting proteins reveals subfunctionalization for defense against fungi and insects. Plant Physiol. 135, 2424–2435. doi: 10.1104/pp.104.044644, PMID: 15299124 PMC520809

[B27] De Lorenzo G. D’Ovidio R. Cervone F. (2001). The role of polygalacturonase-inhibiting proteins (PGIPs) in defense against pathogenic fungi. Annu. Rev. Phytopathol. 39, 313–335. doi: 10.1146/annurev.phyto.39.1.313, PMID: 11701868

[B28] De Lorenzo G. Ferrari S. (2002). Polygalacturonase-inhibiting proteins in defense against phytopathogenic fungi. Curr. Opin. Plant Biol. 5, 295–299. doi: 10.1016/S1369-5266(02)00271-6, PMID: 12179962

[B29] Derbyshire M. C. Raffaele S. (2023). Till death do us pair: Co-evolution of plant–necrotroph interactions. Curr. Opin. Plant Biol. 76, 102457. doi: 10.1016/j.pbi.2023.102457, PMID: 37852141

[B30] Di Matteo A. Bonivento D. Tsernoglou D. Federici L. Cervone F. (2006). Polygalacturonase-inhibiting protein (PGIP) in plant defence: a structural view. Phytochemistry 67, 528–533. doi: 10.1016/j.phytochem.2005.12.025, PMID: 16458942

[B31] Di Matteo A. Federici L. Mattei B. Salvi G. Johnson K. A. Savino C.. (2003). The crystal structure of polygalacturonase-inhibiting protein (PGIP), a leucine-rich repeat protein involved in plant defense. Proc. Natl. Acad. Sci. 100, 10124–10128. doi: 10.1073/pnas.1733690100, PMID: 12904578 PMC187787

[B32] Di Pietro A. Madrid M. P. Caracuel Z. Delgado-Jarana J. Roncero M. I. G. (2003). Fusarium oxysporum: exploring the molecular arsenal of a vascular wilt fungus. Mol. Plant Pathol. 4, 315–325. doi: 10.1046/j.1364-3703.2003.00180.x, PMID: 20569392

[B33] Doehlemann G. Ökmen B. Zhu W. Sharon A. (2017). Plant pathogenic fungi. Fungal Kingdom, 701–726. doi: 10.1128/9781555819583.ch34 PMC11687436 28155813

[B34] Ellur V. (2022). Roles of chickpea polygalacturonase-inhibiting proteins in defense against pathogenic fungi (Washington State University).

[B35] Ellur V. Wei W. Ghogare R. Solanki S. Vandemark G. Brueggeman R.. (2023a). Identification of two novel polygalacturonase-inhibiting proteins (PGIPs) and their genomic reorganization in chickpea (Cicer arietinum). BioRxiv, 2003–2023. doi: 10.1101/2023.03.26.534275 PMC10278945 37342773

[B36] Ellur V. Wei W. Ghogare R. Solanki S. Vandemark G. Brueggeman R.. (2023b). Unraveling the genomic reorganization of polygalacturonase-inhibiting proteins in chickpea. Front. Genet. 14, 1189329. doi: 10.3389/fgene.2023.1189329, PMID: 37342773 PMC10278945

[B37] Elshamy S. S. (2024). Differentiation between Ralstonia solanacearum isolates based on polygalacturonase (PEHA) gene, protein profile and PEHA gene expression. J. Plant Prot. Pathol. 15, 5–10.

[B38] Favaron F. Sella L. D’Ovidio R. (2004). Relationships among endo-polygalacturonase, oxalate, pH, and plant polygalacturonase-inhibiting protein (PGIP) in the interaction between Sclerotinia sclerotiorum and soybean. Mol. Plant-Microbe Interact. 17, 1402–1409. doi: 10.1094/MPMI.2004.17.12.1402, PMID: 15597746

[B39] Federici L. Caprari C. Mattei B. Savino C. Di Matteo A. De Lorenzo G.. (2001). Structural requirements of endo polygalacturonase for the interaction with PGIP (polygalacturonase-inhibiting protein). Proc. Natl. Acad. Sci. 98, 13425–13430. doi: 10.1073/pnas.231473698, PMID: 11687632 PMC60887

[B40] Federici L. Di Matteo A. Fernandez-Recio J. Tsernoglou D. Cervone F. (2006). Polygalacturonase inhibiting proteins: players in plant innate immunity? Trends Plant Sci. 11, 65–70. doi: 10.1016/j.tplants.2005.12.005, PMID: 16406303

[B41] Gadre R. V. Van Driessche G. Van Beeumen J. Bhat M. K. (2003). Purification, characterisation and mode of action of an endo-polygalacturonase from the psychrophilic fungus Mucor flavus. Enzyme Microbial Technol. 32, 321–330. doi: 10.1016/S0141-0229(02)00291-0

[B42] Gomathi V. Gayathri S. Anupama B. Silva J. A. T. Gnanamanickam S. S. (2006). Molecular aspects of polygalacturonase-inhibiting proteins (PGIPs) in plant defense. Floriculture Ornamental Plant Biotechnol. 3, 373–379.

[B43] Govrin E. M. Levine A. (2000). The hypersensitive response facilitates plant infection by the necrotrophic pathogen Botrytis cinerea. Curr. Biol. 10, 751–757. doi: 10.1016/S0960-9822(00)00560-1, PMID: 10898976

[B44] Guo J. Jiang L. Yu A. Han B. Liu A. (2025). Characterization and evolutionary analyses reveal differential selection pressures on PGIc and PGIp during domestication in castor bean. Horticulturae 11, 569. doi: 10.3390/horticulturae11060569

[B45] Hegedus D. D. Rimmer S. R. (2005). Sclerotinia sclerotiorum: when “to be or not to be” a pathogen? FEMS Microbiol. Lett. 251, 177–184. doi: 10.1016/j.femsle.2005.07.040, PMID: 16112822

[B46] Hou W. Mu J. Li A. Wang H. Kong L. (2015). Identification of a wheat polygalacturonase-inhibiting protein involved in Fusarium head blight resistance. Eur. J. Plant Pathol. 141, 731–745. doi: 10.1007/s10658-014-0574-7

[B47] Hou Y. Wu F. Zhao Y. Shi L. Zhu X. (2019). Cloning and expression analysis of polygalacturonase and pectin methylesterase genes during softening in apricot (Prunus Armeniaca L.) fruit. Scientia Hortic. 256, 108607. doi: 10.1016/j.scienta.2019.108607

[B48] Howell J. T. Davis M. R. (2005). Plant defense mechanisms against fungal pathogens: polygalacturonase inhibitor proteins. Can. J. Plant Pathol. 27, 5–15. doi: 10.1080/07060660509507188

[B49] Isshiki A. Akimitsu K. Yamamoto M. Yamamoto H. (2001). Endopolygalacturonase is essential for citrus black rot caused by Alternaria citri but not brown spot caused by Alternaria alternata. Mol. Plant-Microbe Interact. 14, 749–757. 11386370 10.1094/MPMI.2001.14.6.749

[B50] Jayani R. S. Saxena S. Gupta R. (2005). Microbial pectinolytic enzymes: a review. Process Biochem. 40, 2931–2944. doi: 10.1016/j.procbio.2005.03.026

[B51] Jin Y. Zhang Y. Lin L. Ying S. Yu C. (2024). Cucumber PGIP2 is involved in resistance to gray mold disease. Gene 923, 148588. doi: 10.1016/j.gene.2024.148588, PMID: 38763363

[B52] Jones T. M. Anderson A. J. & Albersheim P. (1972). Host-pathogen interactions IV. Studies on the polysaccharide-degrading enzymes secreted by Fusarium oxysporum f. sp. lycopersici. Physiol. Plant Pathol. 2, 153–166. doi: 10.1016/0048-4059(72)90023-9

[B53] Jones J. D. G. Dangl J. L. (2006). The plant immune system. Nature. 444 (7117), 323–329. doi: 10.1038/nature05286, PMID: 17108957

[B54] Jun W. A. Shi F. E. Baohui L. U. Lina N. A. Xue W. A. Zhang Y.. (2022). Fusarium oxysporum f. sp. ginseng, a new forma specialis causing Fusarium root rot of Panax ginseng. Phytopathol. Mediterr. 61, 417–429.

[B55] Kalunke R. M. Tundo S. Benedetti M. Cervone F. De Lorenzo G. D’Ovidio R. (2015). An update on polygalacturonase-inhibiting protein (PGIP), a leucine-rich repeat protein that protects crop plants against pathogens. Front. Plant Sci. 6, 146. doi: 10.3389/fpls.2015.00146, PMID: 25852708 PMC4367531

[B56] Kubicek C. P. Starr T. L. Glass N. L. (2014). Plant cell wall–degrading enzymes and their secretion in plant-pathogenic fungi. Annu. Rev. Phytopathol. 52, 427–451. doi: 10.1146/annurev-phyto-102313-045831, PMID: 25001456

[B57] Kumar S. Dehury B. Tandon G. Jaiswal S. Iquebal M. A. Ahmed K.. (2020). An insight into molecular interaction of PGIP with PG for banana cultivar. Front. Bioscience-Landmark 25, 335–362., PMID: 31585892 10.2741/4809

[B58] Kumar R. Pandey R. Purwar S. Mishra M. K. Rai A. Singh C. M. (2024). Genome-wide identification and characterization of PGIP gene family in Vigna radiata L. Wilczek and its expression in wild non-progenitor, Vigna umbellata L. Thunb. modulate bruchid resistance. J. Plant Biochem. Biotechnol. 34, 1–13. doi: 10.1007/s13562-024-00915-y

[B59] Leckie F. Mattei B. Capodicasa C. Hemmings A. Nuss L. Aracri B.. (1999). The specificity of polygalacturonase-inhibiting protein (PGIP): a single amino acid substitution in the solvent-exposed β-strand/β-turn region of the leucine-rich repeats (LRRs) confers a new recognition capability. EMBO J. 18, 2352–2363. doi: 10.1093/emboj/18.9.2352, PMID: 10228150 PMC1171318

[B60] Lim C. W. Baek W. Jung J. Kim J. H. Lee S. C. (2015). Function of ABA in stomatal defense against biotic and drought stresses. Int. J. Mol. Sci. 16 (7), 15251–15270. doi: 10.3390/ijms160715251, PMID: 26154766 PMC4519898

[B61] Liu C. Q. Hu K. D. Li T. T. Yang Y. Yang F. Li Y. H.. (2017). Polygalacturonase gene pgxB in Aspergillus niger is a virulence factor in apple fruit. PloS One 12, e0173277. 28257463 10.1371/journal.pone.0173277 PMC5336277

[B62] Liu H. Qian M. Song C. Li J. Zhao C. Li G.. (2018). Down-regulation of PpBGAL10 and PpBGAL16 delays fruit softening in peach by reducing polygalacturonase and pectin methylesterase activity. Front. Plant Sci. 9, 1015. doi: 10.3389/fpls.2018.01015, PMID: 30050556 PMC6050397

[B63] Liu N. Sun Y. Wang P. Duan H. Ge X. Li X.. (2018). Mutation of key amino acids in the polygalacturonase-inhibiting proteins Ck PGIP 1 and Gh PGIP 1 improves resistance to Verticillium wilt in cotton. Plant J. 96, 546–561. doi: 10.1111/tpj.14048, PMID: 30053316

[B64] Lorrai R. Ferrari S. (2021). Host cell wall damage during pathogen infection: mechanisms of perception and role in plant-pathogen interactions. Plants 10, 399. doi: 10.3390/plants10020399, PMID: 33669710 PMC7921929

[B65] Lotter H. C. (2010). Characterization and expression of an endopolygalacturonase gene from a lupin anthracnose fungus identified as Colletotrichum lupine VAR. setosum (University of Pretoria).

[B66] Markovič O. Janeček Š. (2001). Pectin degrading glycoside hydrolases of family 28: sequence-structural features, specificities and evolution. Protein Eng. 14, 615–631. doi: 10.1093/protein/14.9.615, PMID: 11707607

[B67] McClelland A. J. Ma W. (2024). Zig, Zag, and’Zyme: leveraging structural biology to engineer disease resistance. Abiotech 5, pp.403–pp.407. doi: 10.1007/s42994-024-00152-w, PMID: 39279864 PMC11399530

[B68] McCombe C. L. Greenwood J. R. Solomon P. S. Williams S. J. (2022). Molecular plant immunity against biotrophic, hemibiotrophic, and necrotrophic fungi. Essays Biochem. 66, 581–593. doi: 10.1042/EBC20210073, PMID: 35587147 PMC9528087

[B69] Murmu S. Rashmi M. Nagrale D. T. Kour T. Singh M. K. Chaurasia A.. (2025). In-silico study of E169G and F242K double mutations in leucine-rich repeats (LRR) polygalacturonase inhibiting protein (PGIP) of Gossypium barbadense and associated defense mechanism against plant pathogens. J. Cotton Res. 8, 3. doi: 10.1186/s42397-024-00203-z

[B70] Nakamura M. Iwai H. Arai K. (2003). Polygalacturonase S31PG1 from Geotrichum candidum citrus race S31 expressed in Schizosaccharomyces pombe versus S31PG2 regarding soft rot on lemon fruit. J. Gen. Plant Pathol. 69, 283–291.

[B71] Nakamura M. Iwai H. (2019). Functions and mechanisms: polygalacturonases from plant pathogenic fungi as pathogenicity and virulence factors. J. Gen. Plant Pathol. 85, 243–250. doi: 10.1007/s10327-019-00856-8

[B72] Naskar A. Roy K. Santra B. Sarkar A. Acharya K. (2023). “An outlook of nematophagous fungi and the underlying mechanism of nematophagy,” in Applied mycology for agriculture and foods (New York: Apple Academic Press), 129–149.

[B73] Noorbatcha I. A. Ismail N. I. Salleh H. M. (2011). Computer aided design of polygalacturonase II from Aspergillus Niger. IIUM Eng. J. 12. doi: 10.31436/iiumej.v12i4.249

[B74] Nühse T. S. (2012). Cell wall integrity signaling and innate immunity in plants. Front. Plant Sci. 3, 280. doi: 10.3389/fpls.2012.00280, PMID: 23248636 PMC3518785

[B75] Palin R. Geitmann A. (2012). The role of pectin in plant morphogenesis. Biosystems 109, 397–402. doi: 10.1016/j.biosystems.2012.04.006, PMID: 22554809

[B76] Pedrolli D. B. Monteiro A. C. Gomes E. Carmona E. C. (2009). Pectin and pectinases: production, characterization and industrial application of microbial pectinolytic enzymes.

[B77] Peng M. de Vries R. P. (2021). Machine learning prediction of novel pectinolytic enzymes in Aspergillus Niger through integrating heterogeneous (post-) genomics data. Microbial Genomics 7, 674. doi: 10.1099/mgen.0.000674, PMID: 34874247 PMC8767319

[B78] Pérez-Donoso A. G. Sun Q. Roper M. C. Greve L. C. Kirkpatrick B. Labavitch J. M. (2010). Cell wall-degrading enzymes enlarge the pore size of intervessel pit membranes in healthy and Xylella fastidiosa-infected grapevines. Plant Physiol. 152, 1748–1759., PMID: 20107028 10.1104/pp.109.148791 PMC2832268

[B79] Petrasch S. (2020). Genetics of strawberry postharvest fruit quality and resistance to necrotrophic fungi (Davis: University of California).

[B80] Poudel D. Yan G. Miranda C. Kreutz G. F. Chowdhury I. A. (2024). Copy number variations at the Rhg1 locus and their relationship with resistance to soybean cyst nematode (Heterodera glycines). Front. Plant Sci. 15, 1504932. doi: 10.3389/fpls.2024.1504932, PMID: 39822960 PMC11736665

[B81] Radha A. Sneha R. Kiruthiga R. Priyadharshini P. Prabhu N. (2019). A review on production of polygalacturonase using various organisms and its applications. Asian J. Biotechnol. Bioresource Technol. 5, 1–12. doi: 10.9734/AJB2T/2019/v5i330063

[B82] Rai D. Siddiqui S. Singh A. K. Pandey M. K. Singh A. Singh D. P. (2024). A comprehensive review of Sclerotinia stem rot in Indian mustard (Brassica juncea). Int. J. Plant Environ. 10, 107–113.

[B83] Ragupathy R. Jolley K. A. Zamuner C. Jones J. B. Redfern J. Behlau F.. (2023). Core-genome multilocus sequence typing for epidemiological and evolutionary analyses of phytopathogenic Xanthomonas citri. Appl. Environ. Microbiol. 89, e02101–e02122. doi: 10.1128/aem.02101-22, PMID: 37067413 PMC10231234

[B84] Rashmi M. Murmu S. Nagrale D. T. Singh M. K. Behera S. K. Shankar R.. (2024). Dataset on double mutation in PGIP of Glycine max improves defense to PG of Sclerotinia sclerotiorum. Data Brief 54, 110518. doi: 10.1016/j.dib.2024.110518, PMID: 38827253 PMC11141275

[B85] Rathinam M. Rao U. Sreevathsa R. (2020). Novel biotechnological strategies to combat biotic stresses: polygalacturonase inhibitor (PGIP) proteins as a promising comprehensive option. Appl. Microbiol. Biotechnol. 104, 2333–2342. doi: 10.1007/s00253-020-10396-3, PMID: 31989226

[B86] Reignault P. Valette-Collet O. Boccara M. (2008). The importance of fungal pectinolytic enzymes in plant invasion, host adaptability and symptom type. Eur. J. Plant Pathol. 120, 1–11. doi: 10.1007/s10658-007-9184-y

[B87] Rollins J. A. Dickman M. B. (2001). pH signaling in Sclerotinia sclerotiorum: identification of a pacC/RIM1 homolog. Appl. Environ. Microbiol. 67, 75–81. doi: 10.1128/AEM.67.1.75-81.2001, PMID: 11133430 PMC92519

[B88] Safran J. (2021). Characterization of pectin remodelling enzymes from *Arabidopsis thaliana* and *Verticillium dahliae*: from protein structure to processivity (Université de Picardie Jules Verne).

[B89] Safran J. Tabi W. Ung V. Lemaire A. Habrylo O. Bouckaert J.. (2023). Plant polygalacturonase structures specify enzyme dynamics and processivities to fine-tune cell wall pectins. Plant Cell 35, 3073–3091. doi: 10.1093/plcell/koad134, PMID: 37202370 PMC10396364

[B90] Schell M. A. Roberts D. P. Denny T. P. (1988). Analysis of the Pseudomonas solanacearum polygalacturonase encoded by pglA and its involvement in phytopathogenicity. J. Bacteriology 170, 4501–4508. 10.1128/jb.170.10.4501-4508.1988 PMC211482 3049534

[B91] Schuster M. Eisele S. Armas-Egas L. Kessenbrock T. Kourelis J. Kaiser M.. (2024). Enhanced late blight resistance by engineering an EpiC2B-insensitive immune protease. Plant Biotechnol. J. 22, 284. doi: 10.1111/pbi.14209, PMID: 37901958 PMC10826977

[B92] Sella L. Castiglioni C. Roberti S. D’Ovidio R. Favaron F. (2004). An endo-polygalacturonase (PG) of Fusarium moniliforme escaping inhibition by plant polygalacturonase-inhibiting proteins (PGIPs) provides new insights into the PG–PGIP interaction. FEMS Microbiol. Lett. 240, 117–124. doi: 10.1016/j.femsle.2004.09.019, PMID: 15500988

[B93] Sheila A. Siti S. Saifur R. M. Naoto O. Tri J. (2022). Manuka honey reduces the virulence of Pectobacterium brasiliense by suppressing genes that encode plant cell wall-degrading enzymes. ASEAN J. Sci. Technol. Dev. 39, 119–124.

[B94] Shi Q. Zheng K. Li H. Wang B. Liang X. Li X.. (2024). LKLPDA: A low-rank fast kernel learning approach for predicting piRNA-disease associations. IEEE/ACM Trans. Comput. Biol. Bioinf. 21 (6), 2179–2187. doi: 10.1109/TCBB.2024.3452055, PMID: 39213276

[B95] Shi H. Zhu L. Zhou Y. Li G. Chen L. Li X. (2009). A cotton gene encoding a polygalacturonase inhibitor-like protein is specifically expressed in petals. Acta Biochim. Biophys. Sin. 41, 316–324. doi: 10.1093/abbs/gmp020, PMID: 19352547

[B96] Shieh M. T. Brown R. L. Whitehead M. P. Cary J. W. Cotty P. J. Cleveland T. E.. (1997). Molecular genetic evidence for the involvement of a specific polygalacturonase, P2c, in the invasion and spread of Aspergillus flavus in cotton bolls. Appl. Environ. Microbiol. 63, 3548–3552., PMID: 9293005 10.1128/aem.63.9.3548-3552.1997 PMC168660

[B97] Silva C. J. Adaskaveg J. A. Mesquida-Pesci S. D. Ortega-Salazar I. B. Pattathil S. Zhang L.. (2023). Botrytis cinerea infection accelerates ripening and cell wall disassembly to promote disease in tomato fruit. Plant Physiol. 191, 575–590., PMID: 36053186 10.1093/plphys/kiac408 PMC9806607

[B98] Sun W. X. Jia Y. J. Feng B. Z. O’Neill N. R. Zhu X. P. Xie B. Y.. (2009). Functional analysis of Pcipg2 from the straminopilous plant pathogen Phytophthora capsici. Genesis 47, 535–544., PMID: 19422018 10.1002/dvg.20530

[B99] Tek M. I. Calis O. (2022). Mechanisms of resistance to powdery mildew in cucumber. Phytopathol. Mediterr. 61, 119–127.

[B100] Tu T. Li Y. Luo Y. Wang Z. Wang Y. Luo H.. (2018). A key residue for the substrate affinity enhancement of a thermophilic endo-polygalacturonase revealed by computational design. Appl. Microbiol. Biotechnol. 102, 4457–4466. doi: 10.1007/s00253-018-8948-y, PMID: 29594344

[B101] Upadhyay S. K. (2024). Defense-related proteins in plants (Elsevier).

[B102] van den Brink J. de Vries R. P. (2011). Fungal enzyme sets for plant polysaccharide degradation. Appl. Microbiol. Biotechnol. 91, 1477–1492. doi: 10.1007/s00253-011-3473-2, PMID: 21785931 PMC3160556

[B103] Varland S. Osberg C. Arnesen T. (2015). N-terminal modifications of cellular proteins: the enzymes involved, their substrate specificities and biological effects. Proteomics 15, 2385–2401. doi: 10.1002/pmic.201400619, PMID: 25914051 PMC4692089

[B104] Volpi C. Raiola A. Janni M. Gordon A. O’Sullivan D. M. Favaron F.. (2013). Claviceps purpurea expressing polygalacturonases escaping PGIP inhibition fully infects PvPGIP2 wheat transgenic plants but its infection is delayed in wheat transgenic plants with increased level of pectin methyl esterification. Plant Physiol. Biochem. 73, 294–301. doi: 10.1016/j.plaphy.2013.10.011, PMID: 24184449

[B105] Xiao Y. Sun G. Yu Q. Gao T. Zhu Q. Wang R.. (2024). A plant mechanism of hijacking pathogen virulence factors to trigger innate immunity. Science 383, 732–739. doi: 10.1126/science.adj9529, PMID: 38359129

[B106] Xu J.-J. Zhou J. Cai Z. Sun J.-L. Li Y.-Z. Fan X.-W. (2024). ZmPGIP1 regulates stem strength by enhancing lignin and cellulose biosynthesis in Arabidopsis thaliana. Biotechnol. Biotechnol. Equip. 38, 2356867. doi: 10.1080/13102818.2024.2356867

[B107] Yang L. (2006). The roles of polygalacturonase-inhibiting proteins in tomato fruit interactions with the grey mold pathogen, Botrytis cinerea (Davis: University of California).

[B108] Yang Y. Lu L. Sun D. Wang J. Wang N. Qiao L.. (2021). Fungus polygalacturonase-generated oligogalacturonide restrains fruit softening in ripening tomato. J. Agric. Food Chem. 70, 759–769. doi: 10.1021/acs.jafc.1c04972, PMID: 34932342

[B109] Zhang T. Sun X. Xu Q. Candelas L. G. Li H. (2013). The pH signaling transcription factor PacC is required for full virulence in Penicillium digitatum. Appl. Microbiol. Biotechnol. 97, 9087–9098., PMID: 23917633 10.1007/s00253-013-5129-x

[B110] Zhu Y. Wu C. Deng Y. Yuan W. Zhang T. Lu J. (2024). Recent advances in virulence of a broad host range plant pathogen Sclerotinia sclerotiorum: A mini-review. Front. Microbiol. 15, 1424130., PMID: 38962122 10.3389/fmicb.2024.1424130 PMC11220166

[B111] Zuppini A. Navazio L. Sella L. Castiglioni C. Favaron F. Mariani P. (2005). An endopolygalacturonase from Sclerotinia sclerotiorum induces calcium-mediated signaling and programmed cell death in soybean cells. Mol. Plant-Microbe Interact. 18, 849–855. doi: 10.1094/MPMI-18-0849, PMID: 16134897

